# Influences of Fe Content and Cold Drawing Strain on the Microstructure and Properties of Powder Metallurgy Cu-Fe Alloy Wire

**DOI:** 10.3390/ma16145180

**Published:** 2023-07-23

**Authors:** Xiaobo Yuan, Ping Zhang, Jianxiang Wang, Biaobiao Yang, Yunping Li

**Affiliations:** 1 State Key Lab for Powder Metallurgy, Central South University, Changsha 410083, China; yuanxiaobo@csu.edu.cn (X.Y.); 223311073@csu.edu.cn (J.W.); lyping@csu.edu.cn (Y.L.); 2 IMDEA Materials Institute, C/Eric Kandel 2, 28906 Getafe, Spain; 3 Department of Materials Science, Polytechnic University of Madrid, E.T.S. de Ingenieros de Caminos, 28040 Madrid, Spain

**Keywords:** Cu-Fe wire, powder metallurgy, cold drawing, ultimate tensile strength, electrical conductivity, Fe phase

## Abstract

To study the effects of Fe content and cold drawing strain on the microstructure and properties, Cu-Fe alloys were prepared via powder metallurgy and hot extrusion. Scanning electron microscopy was applied to observe the Fe phase, and the ultimate tensile strength was investigated using a universal material testing machine. Alloying with an Fe content below 10 wt.% formed a spherically dispersed Fe phase via the conventional nucleation and growth mechanism, whereas a higher Fe content formed a water-droplet-like Fe phase via the spinodal decomposition mechanism in the as-extruded Cu-Fe alloy. Further cold drawing induced the fiber structure of the Fe phase (fiber strengthening), which could not be destroyed by subsequent annealing. As the Fe content increased, the strength increased but the electrical conductivity decreased; as the cold drawing strain increased, both the strength and the electrical conductivity roughly increased, but the elongation roughly decreased. After thermal–mechanical processing, the electrical conductivity and strength of the Cu-40Fe alloy could reach 51% IACS and 1.14 GPa, respectively. This study can provide insight into the design of high-performance Cu-Fe alloys by tailoring the size and morphology of the Fe phase.

## 1. Introduction

Cu-X alloys, where X is a BCC-type metal element (Nb, Ta, Cr, or Fe), are generally recognized to exhibit both high strength and high electrical conductivity [1,2,3,4,5]. Among these BCC-type metal elements, Fe is the most abundant element in the Earth’s crust. In addition, the plastic flow behavior of Fe is similar to that of Cu; therefore, Cu-Fe alloys can be plastically deformed to high strain levels at room temperature [4,5]. Accordingly, Cu-Fe alloys are widely used in industrial fields [6,7,8,9]. The industrial applications of Cu-Fe wire are mainly in the electronics, electrical, and communication fields, such as wires and cables, where the focus is on strength and conductivity. Nevertheless, the Cu-Fe alloy is an immiscible binary alloy, and liquid phase separation takes place during solidification, especially in Cu-Fe alloys with a high Fe content [10,11]. Therefore, numerous defects are easily generated when casting Cu-Fe components [11]. Furthermore, a coarse Fe phase is also unfortunately inevitable, owing to the low cooling rate during solidification [12,13]. These aspects greatly lower the mechanical properties of Cu-Fe alloys, thus restricting their application.

Many attempts have been made to improve the mechanical performance of Cu-Fe alloys by refining the grain size (i.e., grain boundary strengthening) [14,15], work hardening effect [16,17], and aging treatment (i.e., precipitation strengthening) [8,16]. In addition to the above methods, reducing the size of the Fe phase is another effective strategy to improve the comprehensive performance of Cu-Fe alloys. Pang et al. [7] reported that the ultimate tensile strength (UTS) and electrical conductivity (EC) of a cast Cu-10Fe alloy with an initial Fe phase size of 25 μm were 466 MPa and 63% IACS, respectively. Wang et al. [8] reduced the Fe phase size of the same alloy to 20 μm through dual-melt mixed casting and subsequent mechanical processing, leading to an enhancement in UTS to 608 MPa but a reduction in EC to 54%. The increased strength was due to grain boundary strengthening and dislocation strengthening, while the improved EC but reduced strength upon subsequent annealing could be ascribed to grain growth and reduced dislocation density [4]. Zou et al. [18] introduced a novel casting process with an alternating magnetic field to prepare a Cu-14Fe alloy with a finer Fe phase size of 18 μm. The resultant UTS and EC could reach 605 MPa and 48% IACS, respectively. Recently, Wang et al. [19] prepared a Cu-15Fe alloy via spark plasma sintering (SPS) of atomized powder, refining the Fe phase size to ~2 μm after sintering. This led to a further increase in UTS to 750 MPa but a reduction in EC to 22% IACS.

Powder metallurgy is an effective method to enhance the mechanical performance of Cu-Fe alloys via reducing both the grain size of the Cu matrix and the Fe phase size, but this approach is generally detrimental to the EC. The reduced EC can be mainly attributed to the enhanced interface scattering, impurity scattering, and dislocation scattering [20]. The reduced EC of powder-metallurgy Cu-Fe alloys can be partly ascribed to its finer grain size, which leads to increased interface scattering. Moreover, due to the ultrahigh cooling rate during the atomization of powder, it becomes difficult to precipitate the Fe solute from the powder matrix, giving rise to a greater impurity scattering of electrons. Improving the EC becomes a key issue to improve the comprehensive performance of powder-metallurgy Cu-Fe alloys.

To this end, a combination of cyclic cold drawing and annealing was applied to powder-metallurgy Cu-Fe alloys with various cold drawing strain levels, aimed at enhancing the precipitation of Fe solute from the Cu matrix and improving the electrical conductivity by reducing the Fe impurity scattering of electrons. Therefore, in this study, Cu-Fe alloys with different Fe contents were prepared via powder metallurgy, and rod-shaped embryos were obtained via vacuum sintering. Cu-Fe alloy wires under different cold drawing strains were prepared via cold drawing and annealing treatments. The microstructure, EC, and mechanical properties of the wires with different Fe compositions and after various cold drawing strains were investigated in detail.

## 2. Materials and Methods

**Figure 1:**
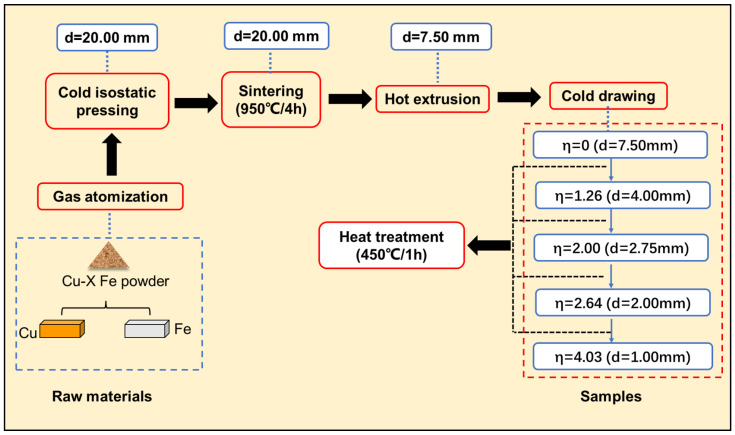
Thermal–mechanical processing flow.

Powders of Cu-5Fe, Cu-10Fe, Cu-20Fe, and Cu-40Fe (wt.%) alloys were prepared via the Ar gas atomization process. The metal powders were placed into a rubber tube, the two ends of which were sealed. The rubber tube containing metal powders was compressed using cold isostatic pressure equipment (LDJ500/1500-300YS, Sichuan Airlines West Sichuan Machinery Co., Ltd., Ya’an, China) to obtain the metal embryos. The pressure of the cold isostatic press was set to 130–150 MPa. The embryos after cold isostatic pressing were subsequently placed into a tubular furnace (SK-G06123K-2R, Kejing Material Technology Co., Ltd., Hefei, China) for sintering under a nitrogen atmosphere containing 4% hydrogen. Sintering was conducted at 950 °C for 4 h, before cooling in the furnace. The embryo body was cylindrical with a length of 90 mm and a diameter of 20 mm. Then, the cylindrical embryos were hot-extruded to obtain rods with a diameter of 7.5 mm. The hot extrusion was performed at 900 °C. The rods (d = 7.5 mm) were drawn into wires with various diameters at room temperature. The drawing equipment used in this experiment was a semiautomatic multi-station pipe-drawing machine (L-B70-A, Shengfei Mechanical Equipment Manufacturing Co., Ltd., Wuxi, China). The stretching mold used in this experiment was a conical mold, and the drawing method was linear horizontal drawing. During cold drawing, the deformation strain is usually defined by the following formula:

(1) $$\eta =ln(A_{0}/A_{f}),$$

where $A_{0}$ is the initial cross-sectional area and $A_{f}$ is the final cross-sectional area. $A_{0}$ in Equation (1) was 43.80 mm^2^ before cold drawing. At cold drawing strains of 0, 1.26, 2.00, 2.64, and 4.03, the wires were taken out for annealing at 450 °C for 1 h to study the microstructure and properties of the Cu-Fe alloys, as schematically shown in Figure 1. An intermediate heat treatment between the cold drawing steps helped to appropriately eliminate a large amount of residual stress stored in the alloy due to deformation, thereby facilitating subsequent cold drawing. Furthermore, Equation (1) was utilized to calculate the deformation strain of the Fe phase (η_Fe_) under various cold drawing strains, by inputting both the initial and the final Fe phase areas. 

A field emission scanning electron microscope (SEM, Quanta 650 FEG, FEI, Boston, MA, USA) was used for the microstructure observation. The surface morphologies of Cu-Fe alloys were observed using the scanning electron microscope through the backscattered electron (BSE) imaging mode, since the atomic numbers of Cu and Fe differ and the two phases can be clearly distinguished under this mode. The instrument used for EC in this work was the QJ36s direct-current low-resistance tester. We measured each sample three times and took the average value as the resistance value (*R*, Ω). Then, we converted the resistance (ρ, Ω·m) into conductivity (*σ*, %IACS), according to the resistivity of annealed pure copper based on the international standard:

(2) $$\sigma =\frac{\rho_{Cu}}{\rho }=\frac{\rho_{Cu}\cdot L}{S\cdot R}$$

where σ is the conductivity (%IACS), $\rho_{Cu}$ is the resistivity of international standard annealed pure copper ($\rho_{Cu}$ = 1.724 Ω·m), ρ is the resistivity of the sample (ρ, Ω·m), R is the resistance value of the sample (*R*, Ω), and *L* and *S* are the length (*L*, m) and cross-sectional area of the tested sample (*S*, m^2^), respectively. Mechanical properties were investigated by using a universal material testing machine (INSTRON 5982, Instron, Norwood, MA, USA). An 80 mm long wire was cut; the upper and lower collets held 30 mm, respectively, leaving 20 mm as the gauge distance; and the strain rate was 1.0 × 10^−3^ s^−1^. The conductivity and ultimate tensile strength were taken as the average of the three measured results.

## 3. Results and Discussion

### 3.1. As-Textured Cu-Fe Alloys

**Figure 2:**
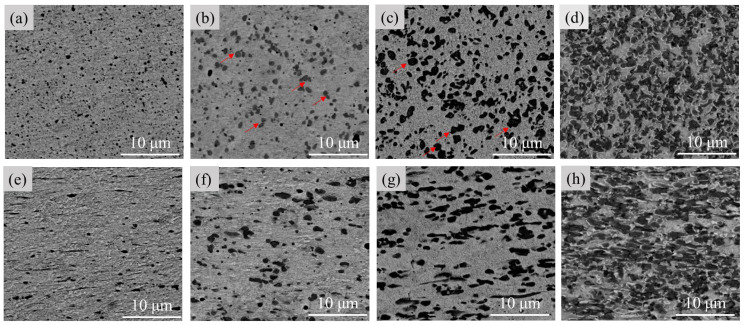
Microstructures of (**a**,**e**) Cu-5Fe, (**b**,**f**) Cu-10Fe, (**c**,**g**) Cu-20Fe, and (**d**,**h**) Cu-40Fe alloys in terms of (**a**–**d**) the cross-section and (**e**–**h**) the longitudinal section. Red arrows are used to point out the location of Fe phase.

**Figure 3:**
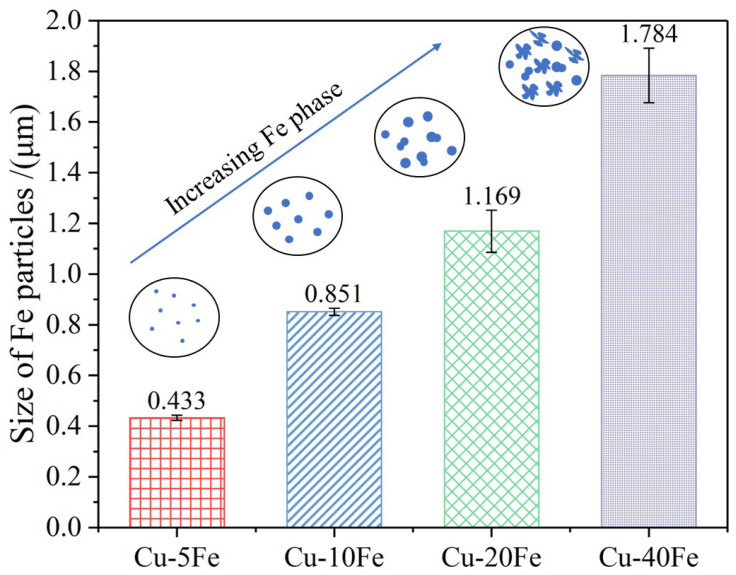
Size of Fe particles in Cu-Fe alloys after the hot extrusion.

Figure 2 shows the microstructures of various Cu-Fe alloys before the cold drawing. From the cross-sectional morphologies, a dark Fe phase is uniformly distributed, and no obvious dendrite structure of the Fe phase is observed in any Cu-Fe alloy. From the longitudinal section, for all alloys, the Fe phase is slightly elongated along the longitudinal direction during hot extrusion. Using Image-J 2.35 software, mean Fe phase sizes in Cu-5Fe, Cu-10Fe, Cu-20Fe, and Cu-40Fe are quantitatively determined to be 0.43, 0.85, 1.07, and 1.78 μm, respectively, as shown in Figure 3. In both Cu-5Fe and Cu-10Fe alloys, the spherical Fe particles are observed to be randomly distributed in the Cu matrix. However, in Cu-20Fe and Cu-40Fe alloys with higher Fe contents, the Fe phase presents irregular shapes such as petal-like or water droplet shapes, which are interconnected together in the Cu matrix. This can be ascribed to the occurrence of phase separation in the liquid phase in Cu-Fe alloys [21,22,23]. 

A large metastable miscibility gap (i.e., bimodal curve) exists in the Cu-Fe binary system above 1200 °C [21]. The liquid phase separation of Cu and Fe has a strong impact on the resultant microstructure of the alloy during the solidification. Shi et al. believed that the concentration of solute elements plays a decisive role in the microstructure of binary alloys with spinodal decomposition [21]. They carried out a phase-field simulation on spinodal decomposition within a liquid droplet. The results showed that when the volume fraction of two phases differs greatly, the minority phase always forms discrete second-phase droplets embedded in the matrix of the major phase; if the volume fraction of the two phases is close, they will form an interpenetrating or bicontinuous morphology, in which each phase forms a continuous interconnection structure. The interconnected structures will break into a series of droplets during further solidification. Their simulation results are in good agreement with the morphologies of the Fe phase in the present work [21].

**Figure 4:**
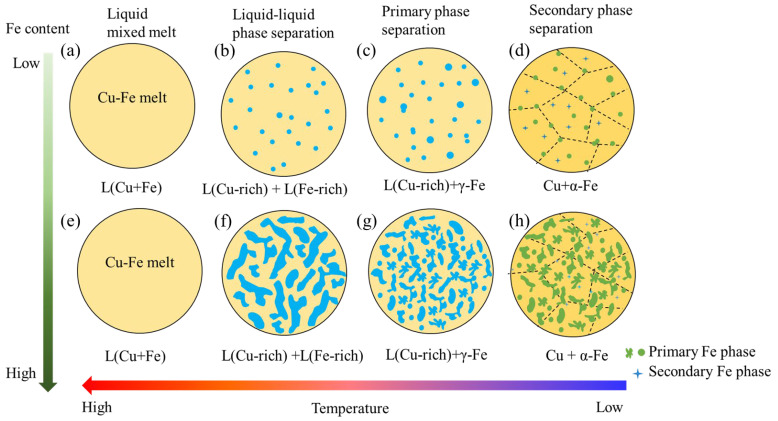
Schematic diagram of the solidification process of Cu-Fe alloys.

A schematic figure describing the liquid phase separation and the resultant microstructure of the Cu-Fe alloy with different Fe contents is given in Figure 4. For the Cu-5Fe, Cu-10Fe, and Cu-20Fe alloys, the volume fraction of the Fe phase is lower in comparison with the Cu matrix (Figure 4a–d). During the liquid–liquid separation stage, the liquid droplets of the Fe phase are dispersed as the core in the Cu matrix (Figure 4b). Owing to the interface energy, some discrete droplets will gather to form a gourd-shaped or slightly larger spherical Fe phase (indicated by the red arrow in Figure 2b,c). Therefore, the Fe phase in the alloy presents a regular spherical dispersion in the Cu matrix (Figure 2a–c) during the process of atomization, where the cooling rate is extremely high. In the Cu-40Fe alloy with a close volume fraction of the Fe phase and Cu phase (Figure 4e–h), the two phases form a continuous interpenetrating structure in the liquid–liquid separation stage. Due to the different surface energy of the two phases, this interconnected structure will break, forming a series of incompletely agglomerated contours (Figure 4h). During the aging process at lower temperature, a small amount of secondary Fe phase precipitates from the Cu matrix (Figure 4d,h) [24,25]. The secondary Fe phase generally exhibits a size ranging from several to dozens of nanometers, which is much finer compared to the primary phase [6,8,9]. Since the fraction of the secondary Fe phase is extremely low, its influence on the main morphology of the Fe phase can be ignored. Therefore, the overall size and morphology of the Fe phase in the Cu-Fe alloy are mainly determined by the primary Fe phase. As shown in Figure 4, in the Cu-Fe alloy with a low Fe content, the phase separation takes place via the conventional nucleation and growth of the Fe phase [26,27]. In the Cu-Fe alloy with a high Fe content, the phase separation is characterized by spinodal decomposition [28,29] in a very short time [10], forming the water-droplet-like Fe phase. 

### 3.2. Cu-Fe Alloys after Cold Drawing and Annealing

**Figure 5:**
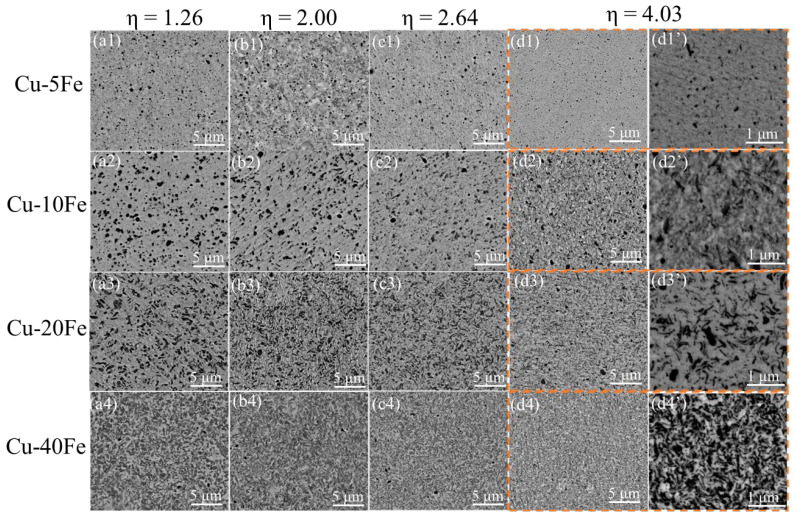
Cross-sectional structures of various Cu-Fe alloys after the cold drawing with various η. The structures of Cu-5Fe, Cu-10Fe, Cu-20Fe, and Cu-40Fe are shown in (**a1**–**d1’**), (**a2**–**d2’**), (**a3**–**d3’**), and (**a4**–**d4’**), respectively. The structures of Cu-Fe alloys under η of 1.26, 2.00, 2.64, and 4.03 are given in (**a1**–**a4**), (**b1**–**b4**), (**c1**–**c4**), and (**d1**–**d4**), (**d1’**–**d4’**), respectively.

**Figure 6:**
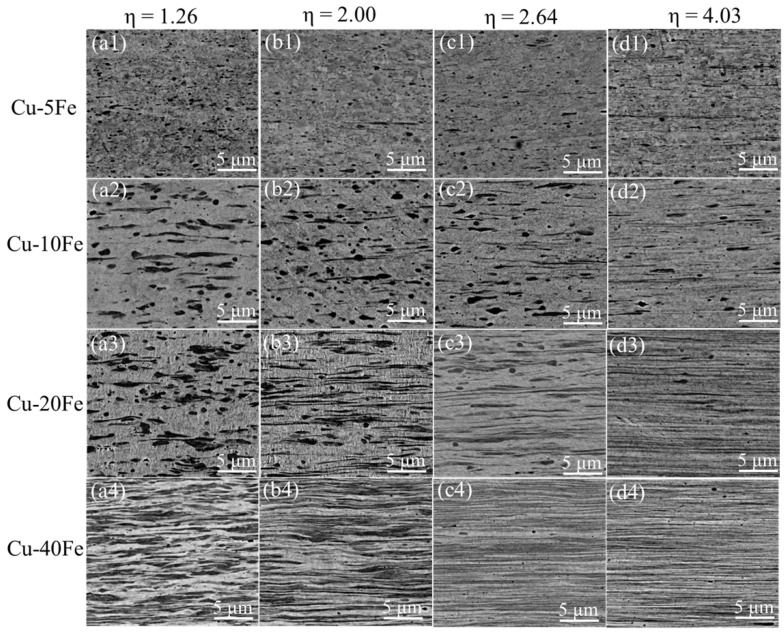
Longitudinal section structures of various Cu-Fe alloys after the cold drawing with various η. The structures of Cu-5Fe, Cu-10Fe, Cu-20Fe, and Cu-40Fe are shown in (**a1**–**d1**), (**a2**–**d2**), (**a3**–**d3**), and (**a4**–**d4**), respectively. The structures of Cu-Fe alloys under η of 1.26, 2.00, 2.64, and 4.03 are given in (**a1**–**a4**), (**b1**–**b4**), (**c1**–**c4**), and (**d1**–**d4**), respectively.

**Figure 7:**
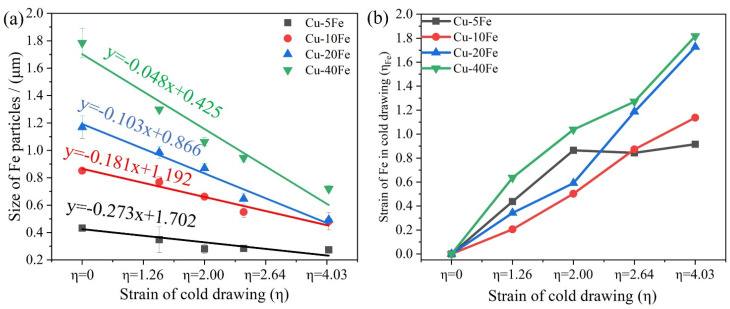
(**a**) The sizes of Fe particles and (**b**) the deformation strain of Fe particles (η_Fe_) in Cu-Fe alloys after the cold drawing with various η.

Figure 5 and Figure 6 show the cross-sectional and longitudinal microstructures of the four Cu-Fe alloys after cold drawing with various η, respectively. From the cross-sectional morphology, with increasing cold drawing strain (η), the Fe phase gradually becomes finer (Figure 5) and is elongated significantly along the drawing direction (Figure 6). Figure 7a quantitatively plots the evolution of the Fe phase size with respect to η. As η increases from 0 to 4.03, the Fe phase in the Cu-40Fe alloy decreases from 1.78 to 0.72 μm. As the Fe content decreases, the reduction rate/slope of the Fe phase size during drawing with respect to the cold drawing strain decreases obviously. The lowest reduction rate of Fe in the Cu-5Fe alloy is observed, i.e., from 0.43 (η = 0) to 0.27 μm (η = 4.03). In addition, the reduction in the Fe phase slows down with the increase in strain. The deformation strain (η_Fe_) of the Fe phase—where the Fe phase in alloys before cold drawing is considered as the initial size—is plotted in Figure 7b with respect to the cold drawing strain. Obviously, under the same cold drawing strain, the η_Fe_ increases with the Fe content, except for Cu5Fe. A possible reason for the abnormal η_Fe_ of the Cu-5Fe alloy is that the initial cross-sectional area of the Fe phase is too small to determine the accurate η_Fe_ during cold drawing. Despite that, the deformation of the Fe phase is more severe in the Cu-Fe alloy with a higher Fe content.

Furthermore, another interesting point is that when η > 2, the η_Fe_ of Cu10Fe, Cu20Fe, and Cu40Fe alloys increases sharply (Figure 7b). This is because when the cold drawing strain is high enough, the Fe phase will fracture into several parts, greatly refining the size of the Fe phase and leading to the increase in η_Fe_. Note that during the cold drawing, both plastic and fracture deformations may occur for the Fe phase, but which one is the dominant deformation mode depends on the morphology of the Fe phase to some extent. To be specific, for the Cu5Fe alloy, the Fe phase is regularly spherical; thus, it will extend uniformly along the drawing direction via the plastic deformation during the drawing process. However, for Cu20Fe and Cu40Fe alloys, the Fe phase is mostly presented in irregular-water-droplet and interconnected shapes; thus, it will tend to fracture during the drawing process. Typical experimental evidence can be found in Figure 6(b4,c3), where an obvious necking phenomenon takes place along the elongation direction of Fe-phase fibers. It must be mentioned that plastic deformation can reduce the Fe fiber spacing and fracture deformation can decrease the Fe phase size. Stepanov et al. [30] reported that the tensile strength and the average spacing of the Fe fiber conform to the Hall–Patch relationship as [30,31]

(3) $$\sigma_{c}=\sigma_{0}+k\cdot \lambda^{-\frac{1}{2}}$$

where $\sigma_{0}$ is a constant (MPa), usually close to the initial strength of the undeformed alloy [32]; *k* is the coefficient of fiber strengthening (MPa·μm^−1/2^); and *λ* is the spacing between the fibers (μm). 

**Figure 8:**
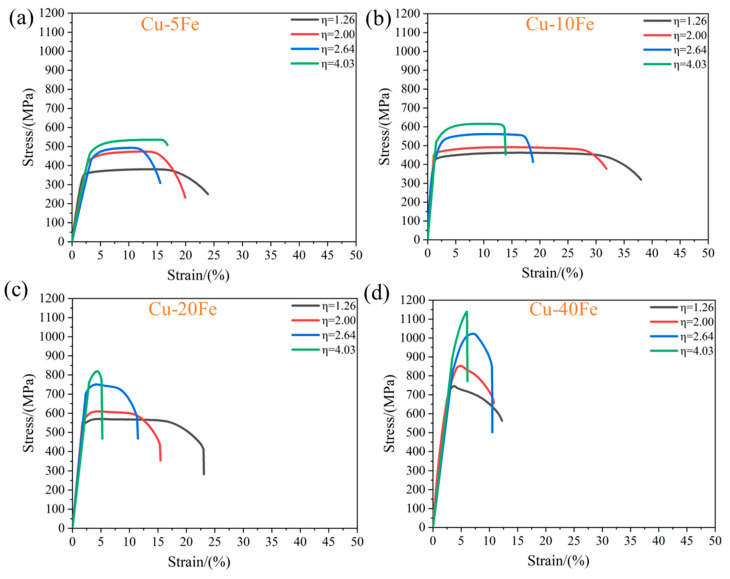
Engineering stress–engineering strain curves of Cu-Fe alloys after cold drawing with various η. (**a**) Cu-5Fe, (**b**) Cu-10Fe, (**c**) Cu-20Fe, and (**d**) Cu-40Fe.

**Figure 9:**
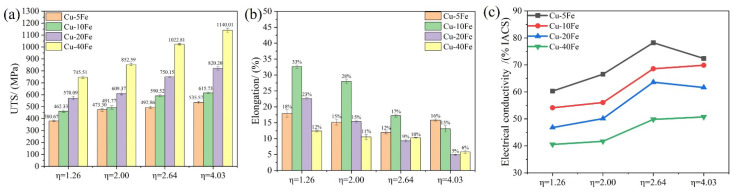
(**a**) The ultimate tensile strength (UTS), (**b**) elongation, and (**c**) electrical conductivity (EC) of various Cu-Fe alloys after cold drawing with various η.

The tensile stress–strain curves of four Cu-Fe alloys with various η are shown in Figure 8, and the UTS, elongation, and electrical conductivity of the alloys are summarized in Figure 9. With the increase in η, for each Cu-Fe alloy, the UTS increases but the elongation decreases (Figure 9a), which could be ascribed to the reduced Fe fiber spacing and the refined Fe phase size. Note that with the increase in Fe content, the effect of fiber reinforcement is more obvious. That means that the Cu-Fe alloy with a higher Fe content is more suitable for the cold drawing deformation to improve mechanical strength. The Cu-10Fe alloy exhibits the largest elongation among all Cu-Fe alloys (Figure 9b). This may be correlated with the unique morphology of the Fe phase, i.e., slender fibers (Figure 6(d2)), which exhibits a good interface with the Cu matrix. Instead, it is difficult for the spherical or ellipsoidal Fe phase in the Cu-5Fe alloy to deform; thus, cracks may easily nucleate near the interface between the Fe phase and Cu matrix, while irregular-shaped and a vast Fe phase in Cu-20Fe and Cu-40Fe alloys presumably accelerates the propagation of cracks, both leading to the premature cracking of the Cu-Fe alloy. Despite that, with the increase in deformation strain, the elongation of the alloys in the tensile test decreases roughly due to more sessile dislocations stored in the alloy. In terms of the electrical conductivity, it increases gradually as the cold drawing strain increases from 0 to 2.64, but changes randomly as the cold drawing strain increases from 2.64 to 4.03 (Figure 9c). 

The dissolved Fe atoms in the Cu matrix can greatly lower the electrical conductivity of alloys. Verhoeven et al. reported that the electrical resistivity increases by 9.2 μΩ/cm for each 1 wt.% Fe alloying in a Cu matrix [4]. For Cu-Fe alloys, the scattering effect of Fe solute in the Cu matrix and the interface between the Fe phase and Cu matrix are the main factors lowering the electrical conductivity. Note that after the cold drawing, the samples are subjected to heat treatment at 450 °C for 1 h, which greatly improves the electrical conductivity of the alloy. A study by Niu et al. demonstrated that the critical temperature of drastic thermal grooving and rapid grain growth for the layered structure of Cu and Fe is 500 °C [33]. This suggests in the study that the annealing cannot damage the Fe fibers; thus, it will not weaken the strengthening effect of Fe fibers. Meanwhile, annealing can improve the electrical conductivity of the alloy, presumably associated with the precipitation process and the dislocation short-circuit diffusion path of solute atoms [34]. The diffusion coefficients of Fe solute atoms during aging are calculated as [35]

(4) $$D_{0}=D_{L}\cdot \left(1-f\right)+D_{P}\cdot f$$

where *D*_0_ is the diffusion coefficient, *D*_L_ is the diffusion coefficient of the lattice, *f* is the fraction of atoms in the dislocation, and *D*_p_ is the diffusion coefficient in the dislocation. The diffusion coefficient of atoms in the dislocation (*D*_p_) is much larger than that in the lattice (*D*_L_). After drawing deformation, the dislocations stored in the alloy provide a fast diffusion channel for the precipitation of Fe atoms. In addition, Li et al. reported that the greater the degree of deformation, the lower the activation energy of solute atom precipitation in the alloy [36]. With the gradual increase in cold drawing strain, the activation energy required for the precipitation of Fe solute atoms in the alloy is reduced gradually and it is easier for the precipitation behavior to occur. Therefore, the electrical conductivity increases with the increase in cold drawing strain (Figure 9c). Note that this argument may not work well at the high cold drawing strain of 4.03 (Figure 9c), presumably because of the large-deformation-induced mechanical alloying of Fe atoms from Fe fibers into the Cu matrix [30].

### 3.3. Comparison of Properties

Ultimate tensile strength and electrical conductivity are important properties for Cu alloys. Synchronously increasing the mechanical strength and electrical conductivity of Cu alloys has been one of the research hotspots in recent decades. Pang et al. reported that the conductivity of a Cu-5Fe alloy prepared by casting and rolling is 63% IACS and the ultimate tensile strength is 466 MPa [7]. Zhang et al. reported that the conductivity of a Cu-30Fe alloy prepared by powder metallurgy and cold rolling was 38% IACS, and the strength was 826 MPa [37]. These, in combination with the previous studies, reveal a simple law: the Cu-Fe alloy with a low Fe content has a high EC but a low UTS, while the Cu-Fe alloy with a high Fe content tends to have a high UTS but a low EC [6,7,18,19,25,32,37,38,39,40,41,42]. 

**Figure 10:**
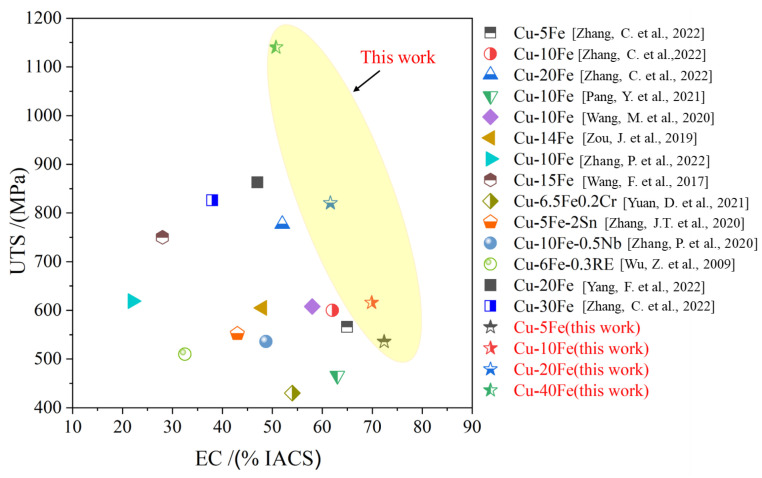
The UTS vs. EC map of the Cu-Fe alloys with different Fe contents [6,7,18,19,25,32,38,39,40,41,42].

In this study, a synchronous improvement in EC and UTS is achieved on the Cu-xFe (x = 5, 10, 20, and 40 wt.%) alloy wires, by the combination of the powder metallurgy technique, hot extrusion, cold drawing, and annealing treatments. For the Cu-5Fe alloy, the EC and UTS reach 72% IACS and 536MPa, respectively, while the EC and UTS of the Cu-40Fe alloy are 51% IACS and 1.14 GPa, respectively, which are obviously higher than those of previously reported Cu-Fe alloys with the same composition (Figure 10). 

## 4. Conclusions

In this study, Cu-xFe (x = 5, 10, 20, and 40 wt.%) alloy wires were prepared by powder metallurgy with the process of cold isostatic pressing, vacuum sintering, hot extrusion, and cold drawing and annealing treatments. The microstructures, mechanical properties, and electrical conductivity of the alloys were analyzed. The conclusions can be drawn as follows:

(1) The Fe phase in the as-extruded Cu-Fe alloys is uniformly dispersed. However, with the increase in Fe content, the Fe phase size increases, and the morphology of the Fe phase transforms from a discrete spheroid to interconnected water droplets, whose formation mechanism is the typical precipitation and spinodal decomposition, respectively.
(2) During the cold drawing, the major deformation mechanism of the Fe phase is plastic deformation for Cu-Fe alloys with a low Fe content, while the combination of plastic and fracture deformations should be the dominant mechanism of the Fe phase for Cu-Fe alloys with a high Fe content. As the cold drawing strain increases, the Fe phase spacing is reduced and the Fe phase size is gradually refined, leading to the gradually enhanced mechanical strength of the alloy.
(3) The combination of the powder metallurgy technique, hot extrusion, cold drawing, and annealing treatments achieves the synchronous improvement in electrical conductivity and mechanical strength of Cu-Fe alloys, which are superior to other reported Cu-Fe alloys.

## Data Availability

Data available on request from the authors.
